# Multigenerational epigenetic inheritance in humans: DNA methylation changes associated with maternal exposure to lead can be transmitted to the grandchildren

**DOI:** 10.1038/srep14466

**Published:** 2015-09-29

**Authors:** Arko Sen, Nicole Heredia, Marie-Claude Senut, Susan Land, Kurt Hollocher, Xiangyi Lu, Mary O. Dereski, Douglas M. Ruden

**Affiliations:** 1 Institute of Environmental Health Sciences, Wayne State University, Detroit, MI 48201; 2 Department of Pharmacology, Wayne State University, Detroit, MI 48201; 3 C. S. Mott Centre for Human Growth and Development, Wayne State University, Detroit, MI 48201; 4 Department of Obstetrics and Gynecology, Wayne State University, Detroit, MI 48201; 5 Union college, 807 Union St, Schenectady, NY 12308; 6 Department of Biomedical Sciences, Oakland University William Beaumont School of Medicine, Rochester, MI 48309.

## Abstract

We report that the DNA methylation profile of a child’s neonatal whole blood can be significantly influenced by his or her mother’s neonatal blood lead levels (BLL). We recruited 35 mother-infant pairs in Detroit and measured the whole blood lead (Pb) levels and DNA methylation levels at over 450,000 loci from current blood and neonatal blood from both the mother and the child. We found that mothers with high neonatal BLL correlate with altered DNA methylation at 564 loci in their children’s neonatal blood. Our results suggest that Pb exposure during pregnancy affects the DNA methylation status of the fetal germ cells, which leads to altered DNA methylation in grandchildren’s neonatal dried blood spots. This is the first demonstration that an environmental exposure in pregnant mothers can have an epigenetic effect on the DNA methylation pattern in the grandchildren.

Studies have suggested that exposure to heavy metal toxicants can influence the global DNA methylation profile. Wright and colleagues have shown significant inverse correlation between patella bone Pb levels and global DNA methylation of LINE1 repeat elements in umbilical cord blood (UCB), suggesting that methylation might serve as a marker for past Pb exposure^1^. Study of global expression patterns and their correlation with DNA methylation in a mouse model of prenatal exposure to Pb have revealed significant association between increase in DNA methylation and transcriptional repression of genes associated with immune response, metal binding, metabolism and transcription/transduction coupling^2^. In recent studies conducted in our lab we have demonstrated that Pb-exposure can cause locus specific changes in DNA methylation which can be detected in dried blood spots (DBS)^3^ and in human embryonic stem cells (hESCs)^4^.

Inheritance of locus-specific DNA methylation changes and its association with environmental exposure has been extensively studied in animal models. For example, Wolff *et al.*, 1998 showed that feeding pregnant *Agouti*^viable yellow^ (*A*^vy^) mice a diet rich in methyl donors correlates with the hyper-methylation of the *Intracisternal-A-Particle* (IAP) transposable element and thereby influences the coat-color phenotype of the offspring^5^. In a recent study, Skinner and colleagues reported differences in the DNA methylation status of genes associated with gonadal sex determination and testis formation of F_3_ male rats from F_0_ rats exposed during gestation to a fungicide, vincozolin^6^. In human cohorts, studies have reported a deterioration in cognitive function and increased incidence of mental illness in children with parents with chronic exposure to smoking, alcohol or environmental toxicants^7^. However, prior to this study, epigenetic effects of environmental exposures beyond one generation have not yet been demonstrated in humans^8^.

Therefore, we tested the hypothesis that human-fetal-germ-cell exposure to environmental toxins causes epigenetic changes in the newborn blood from a grandchild of an exposed pregnant woman (Fig. 1A). During the implantation stage of embryogenesis, most DNA methylation in the embryonic germ cells is erased, including the imprinted genes, so that new imprints can be established depending on the sex of the embryo. During the establishment of the new DNA methylation profile of germ cells, the chromatin is accessible to DNA methyl-transferases (DNMTs), DNA demethylation enzymes such as ten-eleven translocases (TET), and histone modifying enzymes^9^,^10^,^11^. Fetal exposure to environmental toxicants during this process may cause aberrant changes in the DNA methylation and histone modification profiles^4^. In the later developmental stages, the DNA methylation pattern is maintained by the maintenance DNA methyltransferase (DNMT1)^12^. This may contribute to transmission of epigenetic traits from the mother to her grandchildren. For this study, we assume that the locus specific DNA methylation changes due to Pb exposure in the mother was carried over to her grandchildren because she exposed her fetal germ cells during the implantation stage of her pregnancy.

## Results

### Measurement of Lead (Pb) concentration in dried blood spots (DBS)

**Figure 1:**
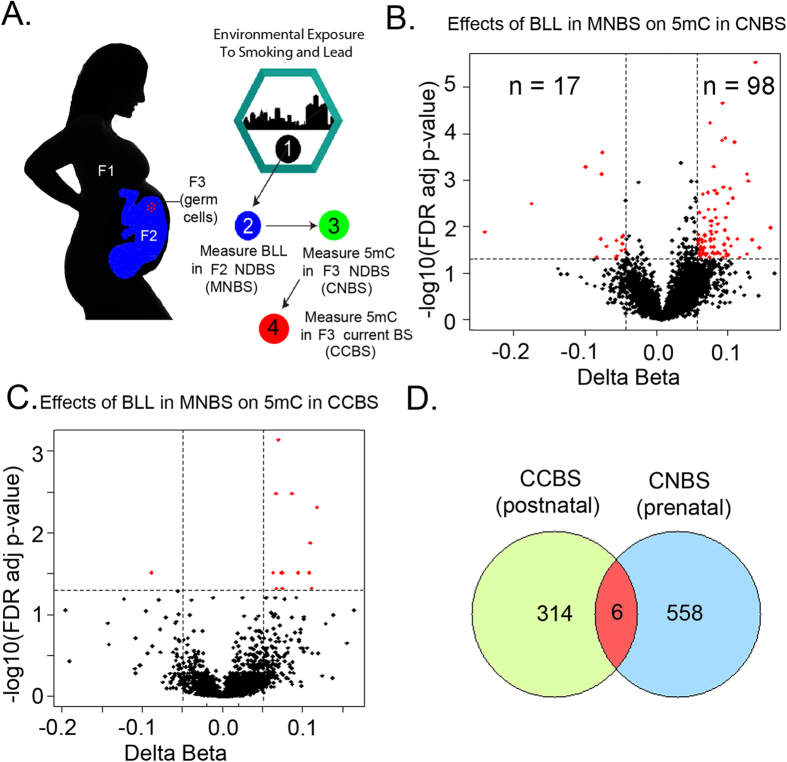
Maternal prenatal exposure to lead (Pb) and its effect on the child’s neonatal and current blood. (**A**) Illustration depicting the plan of study. The F1 represents the maternal grandmother, F2 the mother in this study when she was a fetus, and F3 the child born when the mother matured. (**B**) A-clustering followed by differential methylation analysis by generalized estimating equation (GEE) revealed 115 CpG clusters mapping to 346 CpG sites differentially methylated in child’s neonatal blood spots (CNBS) with high BLL in mother’s neonatal blood spot (MNBS) compared to CNBS with low BLL in MNBS. We observed more hyper-methylated CpG clusters (n = 98) compared to hypo-methylated CpG clusters (n = 17) at an exposure effect cut-off of 0.05 (5%) and an FDR p-value ≤ 0.05. (**C**) Differential methylation analysis revealed no association between DNA methylation levels in a child’s current blood spot (CCBS) and mother’s neonatal BLL (n = 14). (**D**) Overlap between the 320 CpG sites mapping to 116 clusters identified in a previous study^3^, and the 564 CpG sites mapping to 183 clusters identified in this study. The 320 CpG sites (CCBS postnatal) correspond to the effects of BLL in CNBS on CCBS DNA methylation that we reported earlier^3^. The 564 CpG sites (CNBS prenatal) correspond to the effects of BLL in MNBS on CNBS DNA methylation that we report in this study (Fig. 1B). Note children recruited for the previous study are the same as in this study.

In a previous study, we found that acute childhood Pb exposure can alter the methylation profile of DNA isolated from the blood^3^. For this study, we used the same cohort of 35 mother-infant pairs from three Women-Infants-and-Children (WIC) Clinics whom we recruited in Detroit during the summer of 2012. The inclusion criteria was that all of the mothers were born in Michigan after 1984, their children were between 2 and 5 years old at the time of recruitment, and approximately half the children had blood lead levels (BLL) >5 ug/dl in their current blood. BLL was preliminarily measured with a LeadCare II® blood lead testing kit (ESI, Inc.) from a finger prick, and validated later by mass spectrometry (Supplementary Table 1). After obtaining informed consent and IRB approval, we also obtained the neonatal dried bloodspots for both mother and children from the Michigan Neonatal Biobank (www.mnbb.org). BLL was also measured in 3 mm punches from the mothers’ and children’s neonatal dried blood spots by mass spectrometry. Additionally, approximately 1 μg of DNA was isolated from each dried bloodspot and DNA methylation levels at over 450,000 CpG sites were determined with the Illumina Human Methylation 450 K (HM450K) assay (see **Methods**, online). For future reference, we will refer to the child’s neonatal blood spots as CNBS, the mother’s neonatal blood spots as MNBS, and the child’s current blood spots as CCBS (Fig. 1A).

### Contribution of covariates to detectable changes in DNA methylation in dried blood spot (DBS)

We performed two analyses with the DNA methylation data. First, we determined whether high BLLs in the MNBS can significantly affect the methylation profiles of the DNA isolated from the neonatal whole-blood of her children (“Effects of BLL in MNBS on 5 mC in CNBS”; Fig. 1B). Second, we determined whether similar DNA methylation changes occur in the current blood of her children. Using single-value decomposition (SVD) analysis, we explored the contribution of each covariate (maternal BLL, maternal smoking status, gender of offspring, and gestational age) on the DNA methylation profiles of our CNBS samples. SVD does not manipulate the data; rather it calculates the p-values based on the contribution of each component to the variability in the sample. The results indicate that majority of the variation in DNA methylation signal is determined by factors inherent to the HM450K array such as bisulfite conversion rate, hybridization efficiency, and target probe extension (p < 10^−5^) (Supplementary Figure 1A). Among biologically- relevant factors, smoking during pregnancy significantly contributed to the variation (p < 0.01) (Supplementary Figure 1A). This is consistent with previous studies that showed that prenatal tobacco smoke exposure affects global and gene-specific DNA methylation, but they used an earlier version of the HM450K assay that had approximately 27,000 probes^13^. However SVD was unable to detect any significant contribution of other factors such as gender, gestational age of the mothers, or age of the infant or sample BLL in our samples.

Studies have shown that gender can significantly impact the DNA methylation status of autosomal genes^14^. Therefore we looked at gender-specific differences at single-CpG sites in the DNA methylation profile, while controlling for covariates such as child’s neonatal and current BLL levels, mother’s neonatal BLL levels, gestational age, and age of mothers and children, using a mixed-effect model^15^. Similar to what was reported previously^14^, for CNBS, we found 179 autosomal CpG sites with gender-dependent DNA methylation effects at an FDR corrected P-value ≤ 0.05 (Supplementary Figure 1B). For CCBS we found almost the same number of CpG sites N = 144 with gender-dependent DNA methylation effects at mostly the same loci (Supplementary Figure 1C). The numbers of differentially methylated CpG sites associated with gender are modest in CNBS and CCBS suggesting that gender specific differences in DNA methylation are probably not established early in development. Studies have also reported that the DNA methylation levels in whole blood can be confounded by variability in the distribution of the blood-cell-type population^16^. Houseman *et al.*, 2012 proposed a statistical model for estimation of blood-cell-type proportion using HM450K DNA methylation data^16^. We observed a significant variation in estimated granulocyte population across CNBS samples (Supplementary Figure 1D). However, generalized linear model (GLM) analysis showed no significant differences in the blood cell types when the samples were grouped by either the mother’s current smoking status or the mother’s neonatal BLL. This suggests the change in blood cell type population and gender has little contribution toward exposure associated variations in DNA methylation observed in neonates.

### Lead (Pb) exposure dependent changes in DNA methylation profile modeled as CpG clusters

Sofer and colleagues suggested that adjacent CpG sites might show similar behavior in response to environmental exposure^17^. These sites can be assigned to clusters based on the methylation signature (β value) correlation across multiple samples and the pre-specified-base-pair windows. The effect of exposure on these CpG clusters can be tested using linear models such as the generalized-estimating equation (GEE)^17^. Using this powerful strategy for differential methylation analysis, we explored the methylation differences in DNA obtained from CNBS with grandmothers having shown either high BLL (≥5 μg/dl) or low BLL (≤5 μg/dl) during pregnancy (*i.e*., high or low BLL in the MNBS, assuming that the mother’s neonatal BLL corresponds to the grandmother’s BLL). This analysis was performed while controlling for covariates such as sample BLLs, age of the mother, gender of the child, smoking status and gestational age of the mother. At a significance level (FDR p-value < 0.05 and exposure effect cut-off = 0.02), we found 183 CpG clusters mapping to 564 CpG sites which were differentially methylated in CNBS with high BLLs in MNBS compared to low BLL in MNBS (Supplementary Table 2). We observed more hyper-methylated CpG clusters (n = 151) compared to hypo-methylated CpG clusters (n = 32) at an exposure effect cut-off of 0.02 (*i.e*., a 2% change in DNA methylation) (Supplementary Figure 2A). Further increasing the cut-off for differential methylation calls to 0.05 or 5% resulted in significant reduction in identified 5 mC clusters (n = 115). However, the trend in DNA methylation patterns was preserved, with a greater number of hyper-methylated regions (n = 98) compared to hypo-methylated regions (n = 17). A subset of these regions mapped to genes and enhancers which showed significant changes in the DNA methylation status from children (CNBS) who have grandmother’s with high BLL. (Table 1 and Supplementary Figure 3A–D). We note that 65% of our clusters had a change in DNA methylation greater than 5%, and over 10% had a change in DNA methylation over 10% (Supplementary Figure 4).

We were also interested in the Pb-exposure-dependent behavior of CpG sites in the children’s more recently obtained blood (CCBS). We hypothesized that the affected CpG sites in the child’s current blood will eventually re-establish the “normal” DNA methylation levels of the low BLL CCBS. In other words, the effects of acute high BLL on DNA methylation will “normalize” over time, as the blood cells undergo hundreds of mitotic divisions in the absence of Pb. To determine whether fetal germ-line exposure induced epigenetic changes “normalize” during the child’s development, we looked for CpG clusters in the CCBS which are associated with mother’s neonatal BLL, while controlling for covariates such as CCBS BLL, age and gender of the infants, smoking status and gestational age of the mothers. As expected, we found very little correlation between mother’s BLL and CCBS DNA methylation (14 CpG clusters mapping to 37 CpG sites at exposure effect size cut-off of 0.02 or 2%) (“Effects of BLL in MNBS on 5 mC in CCBS”; Fig. 1C). This suggests that, during postnatal development, a child’s changes in DNA methylation returns to the “normal” pattern (*i.e.,* low BLL pattern) within the first 3–5 years of life. We also hypothesized that prenatal and post-natal Pb-exposure will have similar effects on DNA methylation at “Pb-sensitive” CpG sites. In a previous study, on the same cohort, we had identified 116 clusters mapping to 320 CpG sites which were sensitive to acute post-natal exposure to Pb in CCBS, independent of gender at a FDR corrected P-value cut-off of 0.05^3^. We checked for overlap of these sites with the 564 CpG sites which showed statistically significant changes (FDR cut-off 0.05, exposure effect size 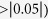 to in-utero exposure to Pb and found only 6 CpG sites in common (Fig. 1D).

### Validation of HM450K array using an *in-vitro* Pb-exposure model.

The detection limit for the HM450K array for calling DNA methylation differences with >95% confidence is ~20% for single CpG sites^18^. We hypothesize that because we are modelling exposure-associated changes as co-regulated clusters containing multiple CpG sites, we can reduce the detection limit to ~10%. The amount of DNA remaining from the blood spots after the HM450K study was not sufficient to perform bisulfite conversion coupled with sequencing. Therefore we decided to use an *in-vitro* model of Pb-exposure in hESCs to validate that the Pb dependent changes ≥10% when modelled as CpG clusters can be detected with some confidence using the HM450K array. We treated H9 embryonic stem cells (hESCs) with 32 μg/dl (1.5 μM) Pb for 24 hours. Then we extracted the DNA and we measured the DNA methylation with HM450K array (our HM450K data for this experiment has been published recently)^4^. We modelled Pb-dependent changes as co-regulated clusters using A-clustering methodology described in this paper. Clusters significant at a FDR corrected p-value cut-off of 0.05 and exposure effect size cutoff of ±10% (N = 279) were selected for further validation. The 279 significant clusters mapped to 716 HM450K probes. Among them 325 CpG probes mapped either inside or near CpG islands. For whole genome validation, we used a standard bisulfite-conversion coupled with shotgun sequencing approach (WGBS) on the same DNA samples that we used for the HM450K studies (EpiGnome™ Kit, Illumina, Inc.). Then we calculated the % methylation within the HM450K cluster windows (n = 279) for Pb-treated samples and non-treated controls. At a minimum 10 reads/cluster, we were able to get significant coverage for only 10/279 CpG clusters (over 1 billion 75 nucleotide single-end reads per treatment were done, see Methods). Approximately half (4/10) of the clusters showed positive correlation between the percent change in DNA methylation with the HM450K and WGBS approaches. A genome browser picture of a representative region is shown (Supplemental Figure 5).

## Discussion

In our study, we provide indirect evidence that Pb-exposure in women during childbirth can affect the locus-specific DNA methylation status of her grandchildren (Fig. 1B). However, the altered DNA methylation profiles of the grandchildren’s blood are apparently “normalized” during post-natal development (Fig. 1C). To determine the significant locus-specific changes in DNA methylation, we used an FDR corrected P-value cut off of 0.05 and an effect size cut off of 5%, which is a standard detection limit of the HM450K array. For example, several studies have reported methylation differences as low as 5%^17^,^19^,^20^,^21^. However, most of these studies have reported differential methylation at single CpG sites. In this paper, we are considering methylation differences in co-regulated regions containing multiple correlated CpG sites. This significantly improves our confidence of detecting true exposure associated changes. At this point, it is worth mentioning, that unlike cancer cells or diseased tissue, where methylation differences are large due to their unnatural cell state, Pb-exposure associated changes in DNA methylation are relatively subtle. Currently, the Human methylation 450K (HM450K) array is the most cost effective assay to study methylation changes with some confidence. Therefore, even if we are focusing on rather small changes (reduced confidence), discounting them completely as noise might result in exclusion of some real biologically relevant epigenetic changes. The main objective of this study is to detect 5 mC changes associated with genes/regulatory regions which may serve as epigenetic biomarkers of transgenerational Pb exposure.

We saw a ~14% increase in DNA methylation in a cluster of 5 probes near the transcription start site (TSS) of Brain Development-Related Molecule 1 or N-Myc Downstream-Regulated Gene (NDRG4) for CNBS with high BLL in MNBS (Supplemental Figure 3A, Table 1). Increase in 5 mC near the TSS might inhibit the binding of transcription factors by recruiting methyl binding proteins such as MECP2, and down-regulate transcription^22^. In mouse models, NDRG4 down-regulation has been associated with reduced levels of Brain Derived Neurotrophic Factor (BDNF), leading to impaired spatial learning and memory^23^. Memory loss and loss of cognitive function is one of the primary disease outcomes of early life Pb exposure, providing some indication that using our approach we are detecting biologically relevant changes in DNA methylation. Moreover, recent studies have used the methylated NDRG4 gene as a candidate biomarker for diagnosis of colorectal cancer (CRC) highlighting its importance in diverse disease processes^24^.

While heavy metal exposure is linked to increases in incidence of cancer in some epidemiology studies^25^, it is premature to link changes in 5 mC in NDRG4 by *in-utero* Pb exposure to increased cancer risk. We saw Pb-exposure associated hypo-methylation (~24% decrease) in the promoter of Nerve Injury Induced Protein 2 (NINJ2) in a small cluster of 2 CpG sites (Table 1, Supplemental Figure 3). NINJ2 has been shown to upregulated in Schwann cells surrounding the distal segment of injured sensory and enteric neurons. Araki *et al.*, 2000 demonstrated that Jurkat cells with stable expression of NINJ2, showed increased aggregation in cell culture, suggesting that NINJ2 functions as a cell adhesion molecule^26^. Co-culture of rat dorsal root ganglion (DRG) neurons with Chinese hamster ovary (CHO) cells expressing NINJ2, showed significant increase in length of the neurites suggesting NINJ2 functions as a promoter of neurite outgrowth in neurodevelopment^26^. Genetic polymorphisms in NINJ2 have also been associated with a decreased risk of Alzeihmer’s disease^27^ and it has been reported to be differentially methylated in patients with borderline personality disorder (BPD)^28^. In our study, NINJ2 showed the most dramatic decrease in 5 mC levels in a promoter region (~24%). As 5 mC levels in the promoter region of genes are negatively correlated with expression, we believe children with grandmothers with high BLL might be overexpressing NINJ2 during development, and this might be a consequence of neuronal injury due to *in-utero* Pb exposure. Therefore, NINJ2 and NDRG4 are our strongest candidates for potential transgenerational biomarkers of Pb exposure for studies in larger cohorts.

We also observed small but significant non-promoter associated increases in DNA methylation in immune system associated genes like Apoliprotein A5 (APOA5) (~5%) (Table 1), and significant demethylation around the promoter of Docking Protein 3 (DOK3) and DEAD (Asp-Glu-Ala-Asp) Box Polypeptide 41(DDX41) (~8%) and transient receptor potential (vanilloid) family member 2 (TRPV2) (~11%) (Table 1, Supplemental Figure 3C). APOA5 knockdown has been shown to significantly improve insulin sensitivity and might be associated with metabolic diseases such as diabetes mellitus^29^. DOK3 has been implicated in the inhibition of lipopolysaccharide signaling in macrophages^30^, while DDX41 has been shown to induce Type1 interferon response on exposure to bacterial infections^31^. TRPV class of receptors contributes to the environmental-sensing properties of neurons and non-sensory cell types such as physiological temperatures, hypo-osmolarity, and mechanical pressure^32^. TRPV2 specifically has been implicated in having a regulatory function in calcium fluxes and pro-inflammatory degranulation events in mast cells^33^. In a recent epidemiological study, increased expression TRPV2 in peripheral blood lymphocytes was also associated with instances of childhood asthma suggesting its role in auto immune processes^34^. Pb exposure has several effects on the immune system, like the ability to shift immune responses away from thymus dependent Th1-associated responses toward Th2-dependent responses^35^, elevate susceptibility to viral and bacterial infections^36^,^37^ and impair the delayed-type hypersensitivity (DTH) response^38^. Therefore, it is not surprising that several genes associated with the immune system show a change in 5 mC in response to *in-utero* Pb exposure, though their utility as transgenerational epigenetic biomarkers require further studies.

A study by Blatter *et al.*, 2014 reported that promoter demethylation is not sufficient to activate gene expression of several genes^39^. In some cases, these changes need to be accompanied by changes in 5 mC level in distal regulatory elements such as enhancers. Therefore, we looked for DMCs located in the vicinity of robust enhancer sites identified in the study by Andersson *et al.*, 2014^40^. At a significance exposure effect cut-off of 5% and FDR p-value of 0.05, we found only 5 DMCs which mapped to less than 300 bps on either side of predicted enhancers (Supplementary Table 3). An example of a significant enhancer has been included in Table 1 and Supplementary Figure 3D. This enhancer was located near a CpG island (chr2:8596907-8597573) and showed an increase of ~9% in its methylation status which may lead to its inhibition. The probes for the HM450K array were designed in context of CpG islands; therefore this array does not have sufficient density to definitively assess the methylation status of a vast majority of regulatory features. However, given this limitation, the lack of methylation changes near enhancer regions suggests that Pb-associated transgenerational changes in methylation are mostly associated with proximal gene regions and not distal regulatory elements like enhancers.

Our preliminary data, within limitations, demonstrates that this novel 2-generational study design might be able to identify the genes which may serve as possible candidate biomarkers for future trans-generational risk assessment studies. Interestingly, we found very little in-utero Pb exposure –associated changes in CpG clusters in CCBS DNA (“Effects of BLL in MNBS on 5 mC in CCBS”; Fig. 1C). This suggests that prenatal in-utero exposure to Pb affects different CpG sites compared to post-natal acute exposure. This also suggests that the epigenetic effect of Pb exposure is dependent on the stage of development during which the exposure occurs and the duration of the exposure. This observation is in agreement with our *in vitro* study in human embryonic stem cells which indicates that acute and chronic exposures to Pb during differentiation into neurons have significantly different effects on the morphology and DNA methylation status than in differentiated neurons^4^.

Unfortunately, the amount of DNA from the neonatal DBS remaining after the HM450K study was not sufficient for validation using whole-genome bisulfite sequencing (WGBS). Therefore, to determine whether a methylation difference of ~10% between Pb-exposed and non-exposed individuals can be detected using the HM450K array, when methylation is modelled as co-regulated clusters, we used DNA from a published Pb-exposure study for validation of the HM450K assay. In our recently published study, we measured the effect of Pb-exposure on the epigenetic profile in an *in-vitro* model of hESC neuronal differentiation^4^. In the previous study, we exposed hESCs to 32 μg/dl of Pb for 24 hours and measured the change in 5 mC with HM450K array^4^. Here, in an attempt to validate the loci which had at least a 10% difference in DNA methylation with the HM450K assay, we used whole genome shotgun bisulfite sequencing (WGBS). Using the analysis paradigm described in the methods section, we were able to validate 4/10 of the regions identified with the HM450K assay which had a minimum coverage depth of 10 (Supplemental Figure 5). We conclude, based on this limited, very expensive and data-intensive WGBS study, that the detection limit for detecting changes as little as 10% with the HM450K assay can achieved if multiple CpG sites in a co-regulated cluster are combined. Currently, we are carrying out pilot studies using over 300,000 padlock-probes^41^ to amplify and validate HM450K cluster from a small quantity of DNA, such as is present in neonatal DBS. If successful, these padlock-studies will be the subject of a future paper.

In conclusion, our pilot study provides indirect evidence that Pb-exposure in women during childbirth can affect the locus-specific DNA methylation status of her grandchildren (Fig. 1B). However, the altered DNA methylation profiles of the grandchildren’s blood are apparently “normalized” during post-natal development. Also, fetal germ-line exposure to Pb apparently has different epigenetic consequences than acute childhood exposure (Fig. 1D). It remains to be determined whether Pb-exposure dependent epigenetic changes are observed in larger and more diverse cohorts, and whether they affect neurodevelopment or other phenotypes associated with high BLL.

## Methods

### Cell culture and treatment

The human ESC line WA09 (H9) was obtained from the WiCell Research Institute (Madison, WI, USA) and maintained in a humidified incubator at 37 °C with 5% CO2, as previously described^4^. Briefly, undifferentiated hESCs (passages 26–39) were cultured in DMEM/F12 supplemented with knockout serum replacement, nonessential amino acids, penicillin/streptomycin, L-Glutamine, 2-mercaptoethanol, and human basic fibroblast growth factor (Life Technologies) on a feeder layer of irradiated mouse embryonic fibroblasts (Globalstem). hESCs were passaged by mechanical dissociation every 4–6 days and their pluripotency frequently tested by immunofluorescence staining for specific markers including Oct4 and Lin28 Stock solutions (100-fold concentrated) of Pb acetate (Pb(C_2_H_3_O_2_)_2_) (Sigma-Aldrich) were prepared in sterile distilled water. Two physiologically relevant concentrations of Pb acetate chosen on the basis of our previous work 24 were tested in this study: 1.5 μM (32 μg/dL). Distilled water was used as a vehicle control. Undifferentiated hESCs were acutely exposed to the different concentrations of Pb or vehicle for 24 hours, at which time the hESC colonies were dissected and their DNA was isolated. Whole genome-bisulfite-sequencing and analysis was done as described (EpiGnome™ Kit, Illumina, Inc.).

### Samples and sample classification

Methods were carried out in accordance with guidelines that were approved by the Wayne State University (WSU) Internal Review Board (IRB), the Michigan Department of Community Health (MDCH) IRB, and the Michigan Neonatal Biobank (MNB) IRB. Experimental protocols were approved by the WSU IRB, the MDCH IRB, and the MNB IRB. Informed consent was obtained from all subjects. For the study, we selected 35 dried blood spots (DBS) collected from mother-infant pairs from Health Fairs ran in three Detroit communities, Rosa Parks, Chene, and Kettering-Butzel, because they have a high prevalence (8–11%) of high BLL in children. The study only included mothers born after January 1, 1987 in Michigan with biological children ages, 3 month to 5 years also born in Michigan. The final sample consisted of 25 male children and 18 female children. We also collected the neonatal DBS and mother neonatal DBS for these mother-infant pairs from the Michigan Neonatal Biobank. Blood Pb measurement was done using Atomic absorption spectroscopy. The corresponding Pb measurement and covariate information can be found in Supplementary Table 1. Methods were carried out in accordance with guidelines that were approved by the Wayne State University Internal Review Board (IRB), the Michigan Department of Community Health (MDCH) IRB, and the Michigan Neonatal Biobank IRB.

### Extraction, shearing and denaturation of DNA

DNA was isolated from dried blood spots with Qiagen EZ1 Advanced® using the DNA Investigator® reagents and protocol card. The “Stains on Fabric” preprocessing and Trace® (tip-dance) instrument protocol was used for isolation. The Quantifiler Human DNA Quantification Kit® (Applied Biosystems, Inc.) was used to determine the amount of amplifiable DNA. Approximately 3 μg genomic DNA was diluted in 130 μl of buffer TE (10 mM Tris (pH 8.0), and 1 mM EDTA (pH 8.0)) and sheared into ~200–600 bp fragment using microcavitation (Covaris, Inc, setting: Duty Cycle = 5%, Intensity = 3, Cycles/burst = 200, Time = 75 seconds run at 6–8 °C). 125 μl of the sheared DNA samples were mixed with 330 μl of buffer TE. The sheared DNA was denatured by boiling it in the Thermomixer at 95 °C and 700 rpm for ten minutes and left on ice for 10 min.

### HM450K bead chip array

The Infinium HM450K array is a high-throughput technology which assesses the methylation status of approximately 450,000 sites on a sample of human genome^42^,^43^. In the Infinium technique, genomic DNA is sheared, denatured, and bisulfite treated. Bisulfite treatment, if applied appropriately, converts cytosine to uracil, but leaves ^5m^C essentially untouched^42^. The DNA is then denatured again and annealed to beads containing DNA oligonucleotides that are complementary to the bisulfite-treated DNA with a 3′ end adjacent to the cytosine whose methylation is being queried. Detection is facilitated by two different probe types (Type 1 and Type 2 probes). The Type 1 probes or the Infinium 1 (Inf1) probes consist of the methylated bead and an un-methylated bead^44^. If the probe for methylated DNA matches the target site there is a single base extension which results in detection which signals into the red channel. Similarly if an un-methylated probe binds to the DNA it signals into the green channel. The type 2 probes or the Infinium 2(Inf2) queries both methylated and unmethylated DNA on a single bead, and the ratio of incorporation of two differently-colored fluorescent nucleotides (Signals A and B) determines the methylation signal. The results are represented in the form of β values, specifically, the average β value (AVG_Beta), representative of the average methylation level of the CpG dinucleotide, and a delta β value which signifies the difference in methylation levels between the control and the experimental group. The Beta or β for the *ith* interrogated CpG nucleotide is:


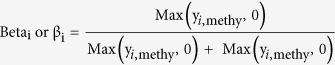


where y_i,methy_ and y_i,unmethy_ are the intensities measured by the *i*^*th*^ methylated and unmethylated probes, respectively. Illumina recommends adding a constant offset α (by default, α = 100) to the denominator to regularize β value when both methylated and unmethylated probe intensities are low. The Beta-value statistic results in a number between 0 and 1, or 0 and 100%^45^. The raw data was retrieved from Genome Studio methylation module version 1.8™ in the form of 2 files; a sample methylation profile and control probe profile. Quality control, signal correction and normalization of the data was carried out using the HM450K BeadChip data processing pipeline proposed by Teschendorff *et al.*, 2013 in R environment (R > 3.0.0)^46^.Several studies have indicated that the Infinium 1 and 2 probes differed in chemistry, henceforth the HM450K are two separate experiment combined as one. The Infinium 1 probes were shown to have a more stable signal and extended dynamic range compared to the Infinium 2 probes^47^. Therefore, a 3-state beta mixture model is utilized to assign methylation values to specific methylation states. Then the probability of assignment to particular state is divided in quantiles and finally a methylation dependent dilation transformation is performed to preserve sample monotonicity^46^. Prior to analysis the beta values were corrected for Batch effect using Combat function in R and potential snp-containing probes were removed from the analysis (>2.15)^48^.

### Statistical analysis

For studying the gender specific effects we CpG association analysis^15^, which analyzes DNA methylation data using a mixed effect model at single CpG site level. For determining the Pb-dependent changes in DNA methylation we used adjacent site clustering algorithm, A-clustering to detect sets of correlated CpG sites and then tested the clusters for multivariate response to environmental exposure to Pb using the generalized estimation (GEE) equation approach. The aforementioned approach is efficiently implemented using the R-package Aclust^17^. For determining the differentially methylated clusters we used the recommended Aclust parameters; Spearman correlation, for calculating the distance between adjacent sites (dist_(i,j)_ = 1 corr_(i,j)_), average clustering type, which require that mean distance between two sites be at least 0.25, 1000 bp distance restriction for merging of clusters, which ensures that clusters located far away from each other are not merged together based on correlation. The clustering approach is implemented with a 999 bp merge initiation step, which clusters all sites wedged between 2 high correlated sites within 999 bps of each other together, to reduce the complexity of data and the analysis time for the Aclust step. Finally the data was analyzed using a generalized estimation equation approach and filtered for significant DMCs using FDR corrected p-value cutoff = 0.05 and exposure effect size 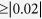. To determine the genomic locations of the probes belonging to individual DMCs, they were annotated using the publicly available Illumina Human Methylation 450k annotation data in R (>2.15). The target genes mapping to DMCs were individually visualized using UCSC genomic browser. The Delta beta or the beta difference between the median of the beta values for each probe for low BLL samples and high BLL samples were mapped by the chromosomal location of the probes. If A-clustering is an effective technique for differentially methylated identification we hypothesized that the change in methylation status visualized using UCSC genome browser and Integrative genome viewer (IGV)will correspond to the exposure effect (i.e. increase or decrease in methylation) predicted by GEE in the respective regions and might serve as a useful tool for visualization. Single value decomposition (SVD) was implemented using the package *ChAMP* and estimation of cell distribution was implemented using the package *minfi*. All statistical analysis was implemented in R. The enhancer regions where downloaded as .*bed* files from Andersson *et al.*, 2014^40^, and the distance from the middle of the robust enhancer sites to the middle of the cluster was calculated using R package ChIPpeakAnno. Then the clusters where subsetted by Maximum distance <301 and exposure effect size 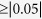 (Supplemental Table 4).

### Analysis of BS-Seq data

The bisulfite sequencing data was aligned to the BS genome using *bismark*^49^. The *.sam files from bismark was used to call 5 mC in CPG context for non-treated and control samples using R package *methylKit*^50^. The methylation calls were further filtered by coverage (min = 10) and possible PCR duplicates were removed by discarding the bases with coverage more than 99.9th percentile of coverage distribution. Then the number of C’s and T’s were calculated for the given HM450K co-regulated clusters. Finally percent (%) methylation for calculated for the clusters to determine the sample specific methylation profile for given regions.

## Additional Information

**Accession codes**: All of the HM450K DNA methylation data are available at the GEO database (accession number GSE67444).

**How to cite this article**: Sen, A. *et al.* Multigenerational epigenetic inheritance in humans: DNA methylation changes associated with maternal exposure to lead can be transmitted to the grandchildren. *Sci. Rep.* **5**, 14466; doi: 10.1038/srep14466 (2015).

## Supplementary Material

Supplementary Information

## Figures and Tables

**Table 1:** Table showing a list of 6 genes/CpG clusters which show Pb dependent change in DNA methylation status in CNBS exposed in-utero to high BLL (high BLL MNBS).

Gene	CpG island	Promoter	Effect size	Standarderror	P-value	FDR	CpG sites/cluster
NDRG4	chr16:58535040-58535596	No (TSS)	0.14	0.04	0.000729	0.028	5
NINJ2	non CpG	Yes	−0.24	0.06	0.000168	0.013	2
TRPV2	non-CpG	Yes	−0.11	0.02	1.29E-06	0.0005	2
DOK3	non-CpG	No (TSS)	−0.08	0.02	4.46E-07	0.00030	4
APOA5	chr11:116661034-116661410	No	0.05	0.01	0.00077	0.029	3
Enhancer	chr2:8596907-8597573	NA	0.090329	0.028656	0.00162	0.0427	2

CpG sites, chromosome (chr) and location of CpG island. Promoter, CpG sites located at the promoter (Yes) or transcription start site (TSS). Effect size, the average change in DNA methylation at the CpG island (e.g., 0.05 is a 5% increase in average DNA methylation). CpG sites/cluster, the number of CpG sites with significant changes in the cluster.
