# Supplementary material for: Multigenerational epigenetic inheritance in humans: DNA methylation changes associated with maternal exposure to lead can be transmitted to the grandchildren
Source: Sci Rep. 2015 Sep 29;5:14466. doi: 10.1038/srep14466 (PMC4586440; doi:10.1038/srep14466)
Supplement: Supplementary Information [file srep14466-s1.pdf]

**Title Page for Supplementary Information:**

**Title of Manuscript:**

# Multigenerational epigenetic inheritance in humans: DNA methylation changes associated with maternal exposure to lead can be transmitted to the grandchildren

**Author List:**

Arko Sen[1, 2], Nicole Heredia[1], Marie-Claude Senut[1], Susan Land[3, 4], Kurt Hollocher[5], Xiangyi Lu[1], Mary O. Dereski[6], and Douglas M. Ruden[1, 3, 4, 7]

1, Institute of Environmental Health Sciences, Wayne State University, Detroit, MI 48201

2, Department of Pharmacology, Wayne State University, Detroit, MI 48201

3, C. S. Mott Centre for Human Growth and Development, Wayne State University, Detroit, MI 48201

4, Department of Obstetrics and Gynecology, Wayne State University, Detroit, MI 48201

5, Union college, 807 Union St, Schenectady, NY 12308

6, Department of Biomedical Sciences, Oakland University William Beaumont School of Medicine, Rochester, MI 48309

7, Corresponding Author: douglasr@wayne.edu (office: 313-577-6688)

**Supplementary Figures**

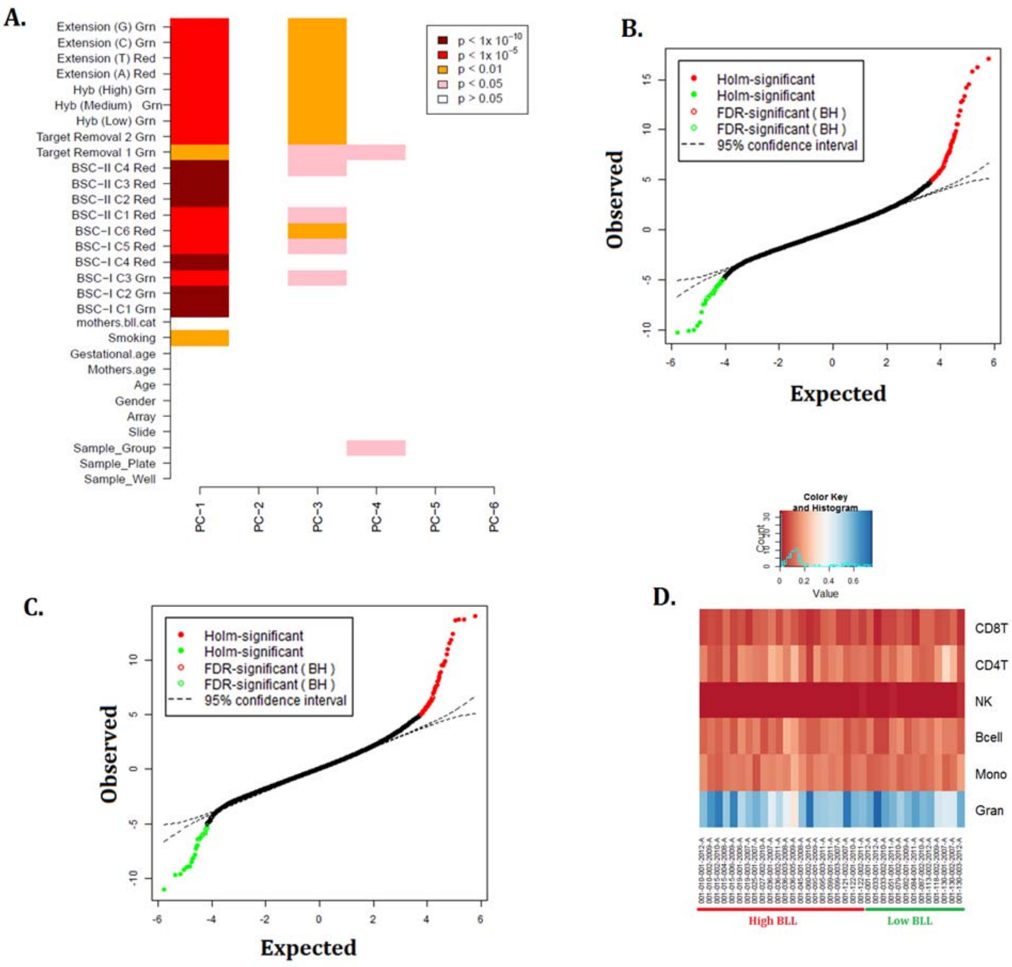


**Supplementary Figure 1:** *Control analysis to account for the effect of covariates such as gender and blood cell distribution.* **A.** P-value associated with each Principle component from a Single value decomposition analysis using ChAMP to determine the effect of each denoted covariate on methylation profile. **B.** T-plot representation of result from the mixed effect model to determine single CpG methylation differences by gender in CNBS by controlling for sample BLL, MNBS BLL, mother's age, gestational age and mother's smoking status. We found 179 CpG sites significantly different by gender at an FDR p-val ≤ 0.05. **C.** T-plot representation of result from mixed effect model to determine single nucleotide differences by gender in CCBS by controlling for sample BLL, MNBS BLL, mother's age, gestational age and mother's smoking status. We found 144 CpG sites significantly different by gender at an FDR p-val ≤ 0.05. **D.** Significant variation in estimated granulocyte population across CNBS samples at predicted by *estimatecellcount* function in *minfi*. However, generalized linear model analysis showed no significant differences when sample where grouped by mother's smoking status or mother's neonatal BLL.

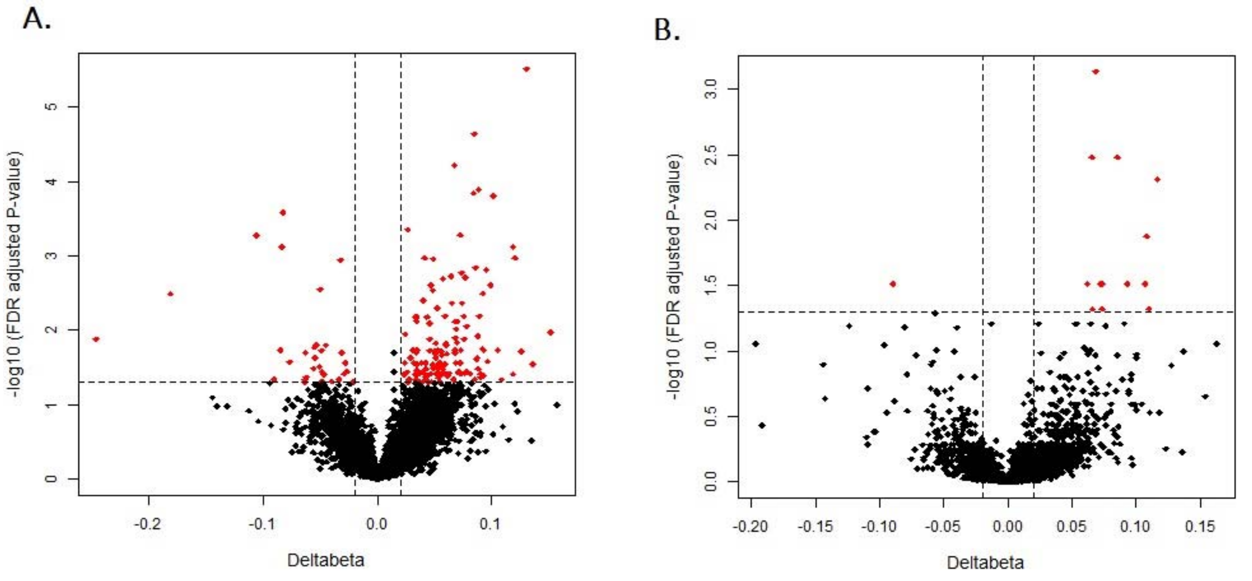


**Supplemental Figure 2:** Differentially methylated sites at a cut-off of 2**%.** A) A-clustering followed by differential methylation analysis by generalized estimating equation (GEE) revealed 183 CpG clusters mapping to 564 CpG sites differentially methylated in child's neonatal blood spots (CNBS) with high BLL in mother's neonatal blood spot (MNBS) compared to CNBS with low BLL in MNBS. We observed more hyper-methylated CpG clusters (n = 151) compared to hypo-methylated CpG clusters (n= 32) at an exposure effect cut-off of 0.02 (2%) and an FDR p-value ≤ 0.05. B) Differential methylation analysis revealed no association between DNA methylation levels in a child's current blood spot (CCBS) and mother's neonatal BLL (n=14).

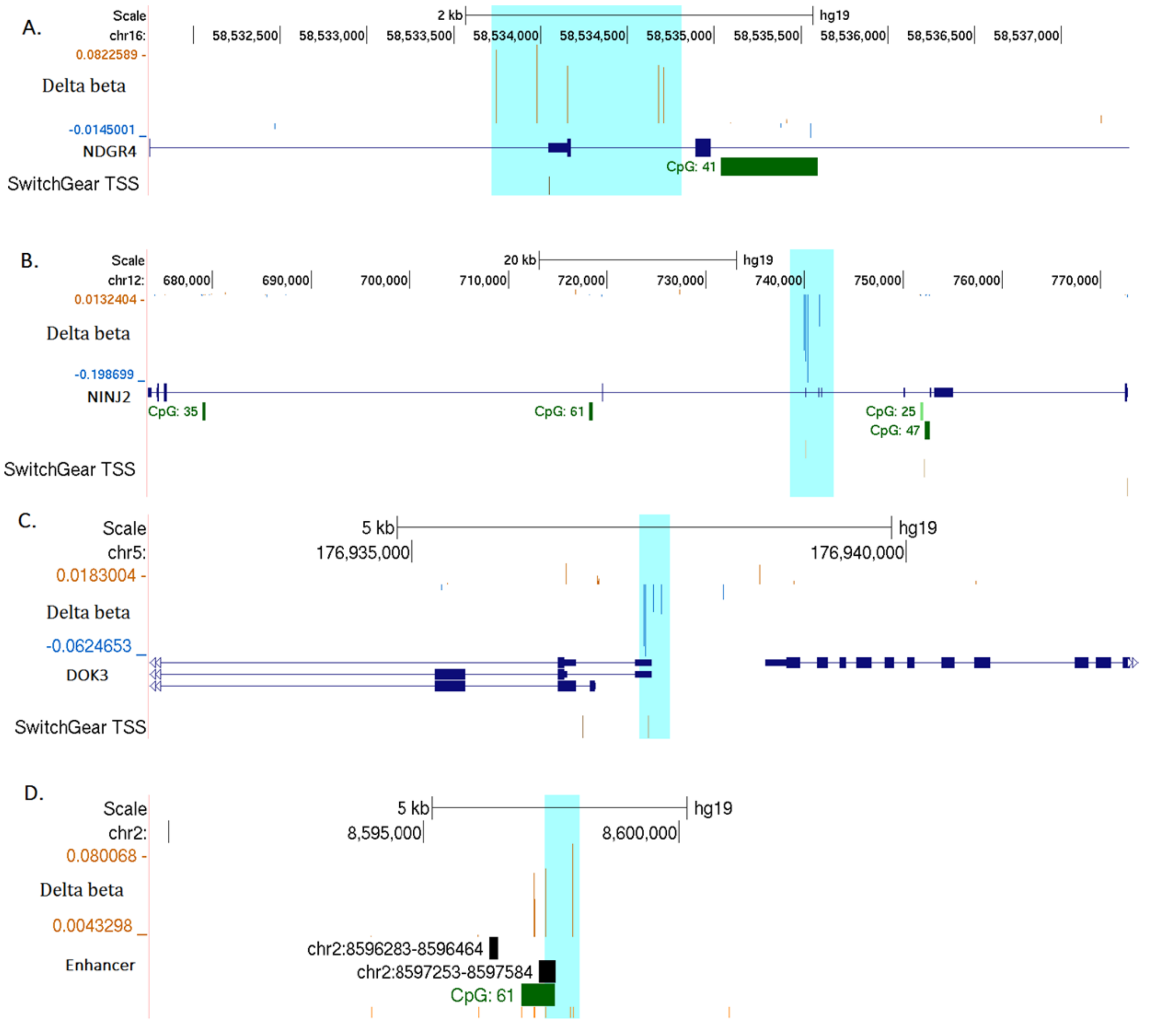


**Suplementary Figure 3:** UCSC genome browser pictures of differentially methylated region detected in CNBS with high BLL in MNBS. The Delta beta or the difference between the mean of the β values for each probe for low BLL samples and high BLL samples were mapped by the chromosomal location of the probes for representative genes A. NDRG4, B. NINJ2, C. DOK3, D.Enhancer identified by Andersson et al, 2014.

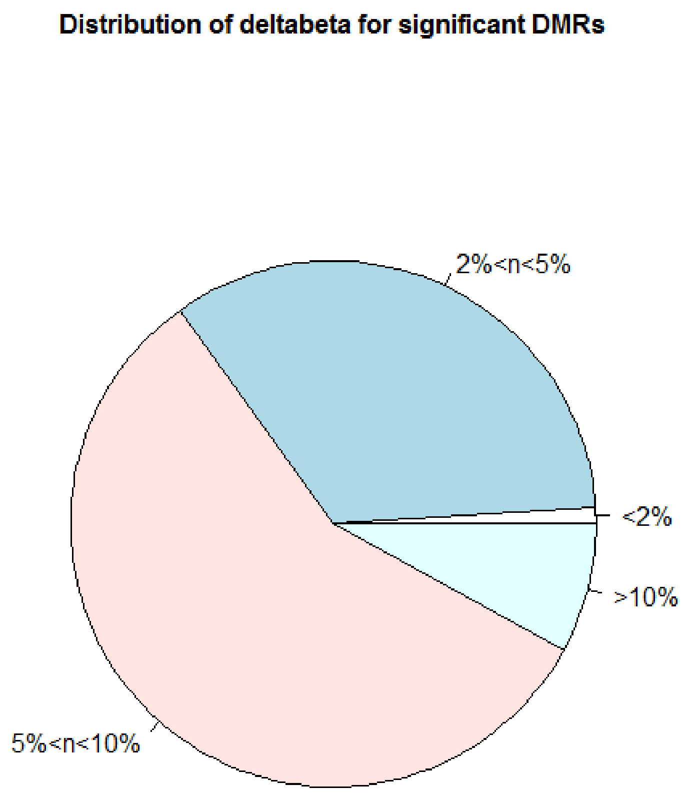


**Supplemental figure 4**: 65% of our significant (FDR p-value ≤ 0.05) differentially methylated clusters had exposure effects ≥ abs (5%) for CNBS with high BLL in MNBS.

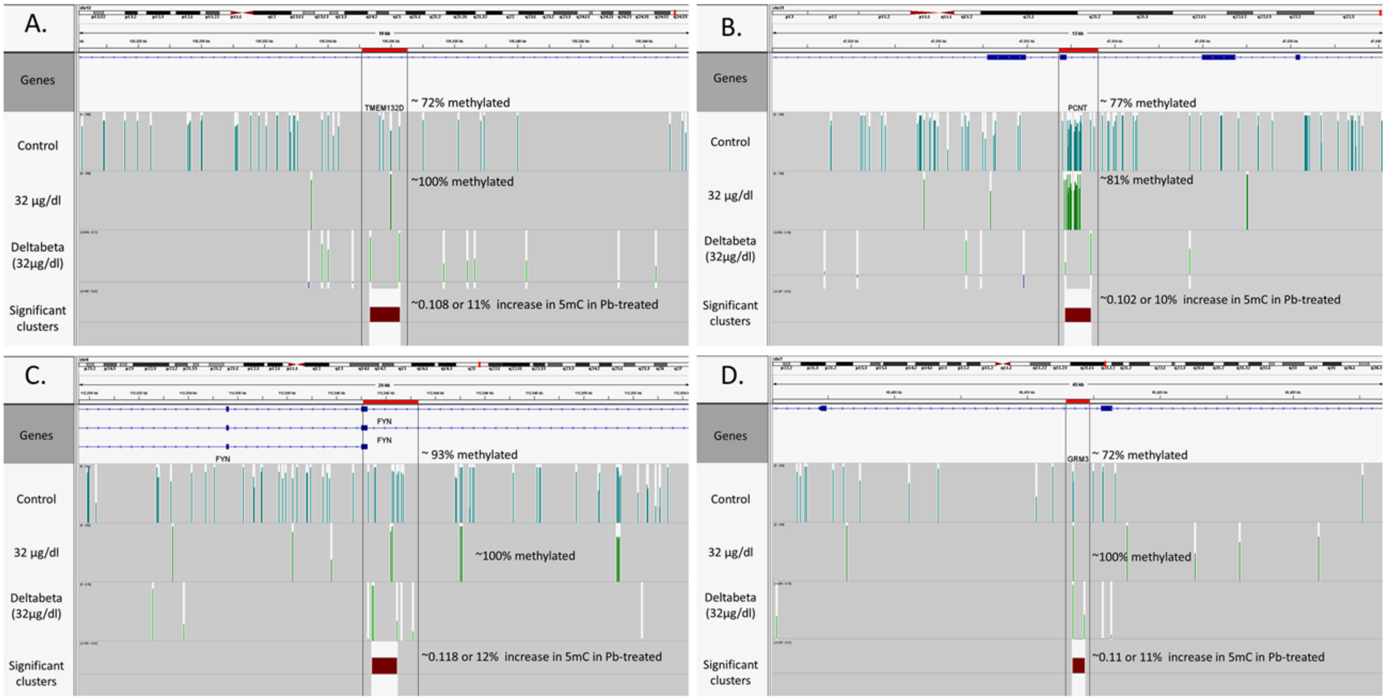


**Supplemental figure 5:** Validation of hM450K using in-vitro studies**.** The Pb –dependent change in 5mC for hESCs exposed to 32 µg/dl of Pb was calculated within co-regulated clusters. Then the number of C's and T's were estimated from BS-Seq data and percent methylation was calculated for the HM450K cluster. The percent methylation for controls (lane1), percent methylation for Pb-exposed hESCs (lane2), single nucleotide difference in 5mC from HM450K (Delta beta, lane3) and cluster effect size from A-clustering analysis of HM450K (Lane4) is illustrated using *Integrative genome viewer* (IGV) for 4 validated regions listed as follows; A) chr12:130335340-130336270, B) chr21:47832880-47833452, C) chr6:112041450-112042446, D) chr7:86413438-86414302.

| Sample | sample bll | mother's bll | Gender | Age (month) | Mother's age (month) | Gestational age (month) | Smoking |
|---|---|---|---|---|---|---|---|
| 001-027-002-2010-B | 0.19009625 | 6.148736497 | F | 24 | 264 | 240 | No |
| 001-001-001-2012-B | 0.579901708 | 2.745941529 | M | 6 | 204 | 186 | No |
| 001-122-001-2010-B | 0.856610711 | 7.29056368 | M | 24 | 240 | 216 | Yes |
| 001-082-001-2009-B | 0.871991444 | 1.378498216 | M | 24 | 216 | 192 | No |
| 001-122-002-2011-B | 1.972338713 | 7.29056368 | F | 12 | 240 | 228 | Yes |
| 001-051-001-2011-B | 2.27245527 | 0.28218839 | M | 12 | 216 | 204 | No |
| 001-087-002-2010-B | 2.938008435 | 4.252003701 | F | 24 | 252 | 228 | Yes |
| 001-060-002-2010-B | 3.035195443 | 36.17606133 | F | 12 | 264 | 252 | Yes |
| 001-130-003-2012-B | 4.378173778 | 3.376888045 | M | 9 | 264 | 243 | No |
| 001-113-002-2012-B | 4.602492159 | 3.011403372 | F | 8 | 228 | 208 | No |
| 001-010-001-2012-B | 4.71525216 | 56.64450079 | M | 5 | 240 | 235 | No |
| 001-036-001-2007-B | 4.735150984 | 11.26302255 | M | 48 | 264 | 216 | Yes |
| 001-033-001-2012-B | 5.098809195 | 2.244241237 | M | 3 | 228 | 225 | No |
| 001-036-005-2009-B | 5.809591329 | 11.26302255 | M | 36 | 264 | 228 | Yes |
| 001-084-001-2011-B | 5.94181757 | 1.768640127 | M | 12 | 216 | 204 | No |
| 001-015-006-2009-B | 6.248038356 | 6.413477367 | F | 36 | 264 | 228 | No |
| 001-025-001-2007-B | 6.436692662 | 5.171338965 | M | 60 | 252 | 192 | No |
| 001-045-001-2008-B | 6.860479927 | 5.987767511 | M | 36 | 252 | 216 | No |
| 001-095-001-2009-B | 7.04519292 | 5.636077432 | M | 36 | 228 | 192 | No |
| 001-121-002-2007-B | 7.206834813 | 6.614628519 | F | 60 | 240 | 180 | Yes |
| 001-099-003-2007-B | 7.430384158 | 7.507768472 | M | 60 | 228 | 168 | No |
| 001-130-001-2007-B | 7.626344312 | 3.376888045 | M | 48 | 264 | 216 | No |
| 001-036-002-2011-B | 8.502613523 | 11.26302255 | F | 12 | 264 | 252 | Yes |
| 001-036-003-2008-B | 9.288905445 | 11.26302255 | M | 48 | 264 | 216 | Yes |
| 001-015-002-2010-B | 9.640403264 | 6.413477367 | F | 36 | 264 | 228 | No |
| 001-015-004-2008-B | 9.665637279 | 6.413477367 | F | 48 | 264 | 216 | No |
| 001-019-001-2006-B | 9.725285685 | 6.999675563 | M | 60 | 276 | 216 | Yes |
| 001-095-003-2011-B | 10.20218454 | 5.636077432 | M | 12 | 228 | 216 | No |
| 001-130-002-2007-B | 10.47605773 | 3.376888045 | F | 48 | 264 | 216 | No |
| 001-099-001-2011-B | 11.04437582 | 7.507768472 | M | 12 | 228 | 216 | No |
| 001-118-002-2009-B | 11.83912714 | 2.722341717 | F | 36 | 252 | 216 | No |
| 001-033-002-2010-B | 16.97667656 | 2.244241237 | F | 24 | 228 | 204 | No |
| 001-019-003-2007-B | 17.22238377 | 6.999675563 | M | 48 | 276 | 228 | Yes |
| 001-010-002-2009-B | 24.20912991 | 56.64450079 | F | 24 | 240 | 216 | No |
| 001-079-002-2010-B | 32.80080749 | 2.671777556 | F | 24 | 252 | 228 | No |
| 001-051-001-2011-A | 1.241657755 | 0.28218839 | M | NA | 216 | 204 | No |
| 001-082-001-2009-A | -0.559041588 | 1.378498216 | M | NA | 216 | 192 | No |
| 001-084-001-2011-A | 1.627906418 | 1.768640127 | M | NA | 216 | 204 | No |
| 001-033-001-2012-A | 2.675718869 | 2.244241237 | M | NA | 228 | 225 | No |
| 001-033-002-2010-A | 4.48833828 | 2.244241237 | F | NA | 228 | 204 | No |
| 001-079-002-2010-A | 4.078826258 | 2.671777556 | F | NA | 252 | 228 | No |
| 001-118-002-2009-A | 1.368164285 | 2.722341717 | F | NA | 252 | 216 | No |
| 001-001-001-2012-A | 0.132033982 | 2.745941529 | M | NA | 204 | 186 | No |
| 001-113-002-2012-A | 0.526838178 | 3.011403372 | F | NA | 228 | 208 | No |
| 001-130-003-2012-A | 1.559798609 | 3.376888045 | M | NA | 264 | 243 | No |
| 001-130-001-2007-A | 1.67457733 | 3.376888045 | M | NA | 264 | 216 | No |
| 001-130-002-2007-A | 4.367599524 | 3.376888045 | F | NA | 264 | 216 | No |
| 001-087-002-2010-A | 1.054781846 | 4.252003701 | F | NA | 252 | 228 | Yes |
| 001-025-001-2007-A | 8.759664027 | 5.171338965 | M | NA | 252 | 192 | No |
| 001-095-003-2011-A | -0.002739693 | 5.636077432 | M | NA | 228 | 216 | No |
| 001-095-001-2009-A | 1.616803451 | 5.636077432 | M | NA | 228 | 192 | No |
| 001-045-001-2008-A | -3.885461602 | 5.987767511 | M | NA | 252 | 216 | No |
| 001-027-002-2010-A | 0.497807044 | 6.148736497 | F | NA | 264 | 240 | No |
| 001-015-002-2010-A | 0.667523822 | 6.413477367 | F | NA | 264 | 228 | No |
| 001-015-006-2009-A | 1.383208565 | 6.413477367 | F | NA | 264 | 228 | No |
| 001-015-004-2008-A | 1.728121508 | 6.413477367 | F | NA | 264 | 216 | No |
| 001-121-002-2007-A | -4.3374629 | 6.614628519 | F | NA | 240 | 180 | Yes |
| 001-019-003-2007-A | 1.497650833 | 6.999675563 | M | NA | 276 | 228 | Yes |
| 001-019-001-2006-A | 3.819612838 | 6.999675563 | M | NA | 276 | 216 | Yes |
| 001-122-001-2010-A | -7.576020476 | 7.29056368 | M | NA | 240 | 216 | Yes |
| 001-122-002-2011-A | -0.034270196 | 7.29056368 | F | NA | 240 | 228 | Yes |
| 001-099-003-2007-A | 2.376179089 | 7.507768472 | M | NA | 228 | 168 | No |
| 001-099-001-2011-A | 9.40974033 | 7.507768472 | M | NA | 228 | 216 | No |
| 001-036-003-2008-A | -0.214657358 | 11.26302255 | M | NA | 264 | 216 | Yes |
| 001-036-005-2009-A | 0.233450692 | 11.26302255 | M | NA | 264 | 228 | Yes |
| 001-036-002-2011-A | 1.617716682 | 11.26302255 | F | NA | 264 | 252 | Yes |
| 001-036-001-2007-A | 2.896384326 | 11.26302255 | M | NA | 264 | 216 | Yes |
| 001-060-002-2010-A | 2.494466541 | 36.17606133 | F | NA | 264 | 252 | Yes |
| 001-010-002-2009-A | 0.587111426 | 56.64450079 | F | NA | 240 | 216 | No |
| 001-010-001-2012-A | 1.04007402 | 56.64450079 | M | NA | 240 | 235 | No |

**Supplementary Table 1:** Covariate and Blood Lead Level (BLL) information used for DNA methylation analysis for 35 samples. Sample names ending with A (Child's Neonatal blood spots), and with B (Child's current blood spots).

**Supplementary Table 2:** Table showing a list of all genes/CpG clusters which show Pb dependent change in DNA methylation status in CNBS exposed in-utero to high BLL (high BLL MNBS) at exposure effect size cut-off > 0.05 or 5% and FDR ≤ 0.05.

| cluster no. | Effect Size | Standard error | P-value | CpG sites/Cluster | CpG sites | FDR |
|---|---|---|---|---|---|---|
| 672 | 0.130861 | 0.02126 | 7.50E-10 | 2 | ;cg20055237;cg05865340 | 3.09E-06 |
| 598 | 0.084901 | 0.014865 | 1.12E-08 | 2 | ;cg16877087;cg23080818 | 2.31E-05 |
| 2823 | 0.067546 | 0.012342 | 4.43E-08 | 2 | ;cg10081469;cg15891218 | 6.08E-05 |
| 3903 | 0.08896 | 0.016827 | 1.24E-07 | 3 | ;cg17250863;cg12419685;cg19481439 | 0.000128 |
| 397 | 0.084224 | 0.01612 | 1.74E-07 | 4 | ;cg20462561;cg23926439;cg23012917;cg25138412 | 0.000143 |
| 536 | 0.102005 | 0.019715 | 2.29E-07 | 4 | ;cg04850045;cg20265528;cg22878388;cg18192325 | 0.000157 |
| 1141 | -0.08219 | 0.016281 | 4.46E-07 | 4 | ;cg12177334;cg01278291;cg08394278;cg07608052 | 0.000262 |
| 696 | 0.02689 | 0.005463 | 8.57E-07 | 8 | ;cg20850873;cg13047869;cg16606324;cg10729496;cg00166722;cg08888203;cg18621852;cg15117754 | 0.000441 |
| 2929 | 0.072807 | 0.01497 | 1.15E-06 | 2 | ;cg20663376;cg13814747 | 0.000528 |
| 3302 | -0.1054 | 0.021772 | 1.29E-06 | 2 | ;cg26719625;cg09093656 | 0.000531 |
| 769 | -0.0831 | 0.01753 | 2.13E-06 | 3 | ;cg04995300;cg17646820;cg23477460 | 0.00076 |
| 3746 | 0.11876 | 0.025093 | 2.22E-06 | 2 | ;cg14942863;cg19556473 | 0.00076 |
| 2080 | 0.041795 | 0.009005 | 3.46E-06 | 2 | ;cg22867608;cg12397802 | 0.00107 |
| 938 | 0.120837 | 0.026093 | 3.64E-06 | 3 | ;cg01777861;cg09978860;cg07123701 | 0.00107 |
| 664 | 0.048945 | 0.010614 | 4.00E-06 | 3 | ;cg14266287;cg08773314;cg03560586 | 0.001096 |
| 758 | -0.0316 | 0.006885 | 4.45E-06 | 2 | ;cg16898425;cg11163571 | 0.001144 |
| 1752 | 0.08614 | 0.019022 | 5.94E-06 | 4 | ;cg09652312;cg24517467;cg09406004;cg25265226 | 0.001438 |
| 260 | 0.095582 | 0.021234 | 6.75E-06 | 4 | ;cg18140268;cg19265480;cg23206461;cg13067553 | 0.001544 |
| 1350 | 0.07407 | 0.016574 | 7.85E-06 | 16 | ;cg15713058;cg23942884;cg00063006;cg22859054;cg02269978;cg24639679;cg19628603;cg00797104;cg11060379;cg17352045;cg00892368;cg21685006;cg18942934;cg13493635;cg15076318;cg08873792 | 0.001702 |
| 149 | 0.064743 | 0.014591 | 9.11E-06 | 3 | ;cg06155303;cg08597839;cg00107241 | 0.001874 |
| 1046 | 0.077498 | 0.017545 | 1.00E-05 | 2 | ;cg18804667;cg05593411 | 0.001961 |
| 3821 | 0.057643 | 0.013099 | 1.08E-05 | 2 | ;cg25594486;cg00380835 | 0.002022 |
| 91 | 0.047352 | 0.010898 | 1.39E-05 | 2 | ;cg17158913;cg04149978 | 0.002473 |
| 375 | 0.099198 | 0.02287 | 1.44E-05 | 2 | ;cg15730491;cg26047066 | 0.002473 |
| 295 | -0.04977 | 0.011573 | 1.71E-05 | 2 | ;cg22500132;cg22531371 | 0.00281 |
| 2370 | 0.048644 | 0.011364 | 1.86E-05 | 5 | ;cg25104637;cg26388730;cg24524285;cg23907108;cg15939287 | 0.00295 |
| 2663 | 0.092634 | 0.021801 | 2.15E-05 | 2 | ;cg13586425;cg25772418 | 0.003228 |
| 1776 | -0.18049 | 0.042528 | 2.20E-05 | 4 | ;cg17920646;cg24536782;cg03116837;cg14317384 | 0.003228 |
| 2364 | 0.040771 | 0.009733 | 2.80E-05 | 2 | ;cg17590162;cg04582871 | 0.003979 |
| 865 | 0.074202 | 0.017869 | 3.29E-05 | 2 | ;cg25607670;cg18025886 | 0.00438 |

| | | | | | | |
|---|---|---|---|---|---|---|
| 773 | 0.065321 | 0.015733 | 3.30E-05 | 2 | ;cg00258809;cg25112202 | 0.00438 |
| 3398 | 0.053011 | 0.012888 | 3.90E-05 | 4 | ;cg15117739;cg23245942;cg04917446;cg12057127 | 0.005016 |
| 4072 | 0.059295 | 0.014673 | 5.32E-05 | 2 | ;cg00549798;cg08362785 | 0.006501 |
| 261 | 0.075658 | 0.018759 | 5.50E-05 | 2 | ;cg00171578;cg10537450 | 0.006501 |
| 1903 | 0.089381 | 0.022168 | 5.53E-05 | 2 | ;cg22258713;cg23193613 | 0.006501 |
| 1274 | 0.044145 | 0.010969 | 5.71E-05 | 3 | ;cg24710951;cg08743794;cg26004235 | 0.006533 |
| 241 | 0.033259 | 0.008284 | 5.94E-05 | 2 | ;cg10806711;cg16255156 | 0.006593 |
| 3002 | 0.034779 | 0.008674 | 6.09E-05 | 6 | ;cg03291024;cg13778201;cg08267442;cg01351822;cg16414568;cg08551047 | 0.006593 |
| 391 | 0.043114 | 0.010783 | 6.38E-05 | 2 | ;cg25734842;cg15033163 | 0.006737 |
| 3399 | 0.034554 | 0.008727 | 7.52E-05 | 2 | ;cg23690166;cg26608174 | 0.00756 |
| 3354 | 0.070167 | 0.017724 | 7.53E-05 | 2 | ;cg26960939;cg07248223 | 0.00756 |
| 192 | 0.068127 | 0.017245 | 7.80E-05 | 2 | ;cg12275060;cg03912703 | 0.00764 |
| 1138 | 0.046181 | 0.011748 | 8.46E-05 | 4 | ;cg01287088;cg06847624;cg00642460;cg20095656 | 0.008101 |
| 2925 | 0.078425 | 0.020093 | 9.50E-05 | 2 | ;cg16276982;cg09214243 | 0.008886 |
| 1408 | 0.069139 | 0.01783 | 0.000105 | 3 | ;cg17962638;cg18288532;cg16218241 | 0.009643 |
| 2199 | 0.151873 | 0.039475 | 0.000119 | 2 | ;cg23771949;cg15175351 | 0.010685 |
| 2118 | 0.024513 | 0.006403 | 0.000129 | 3 | ;cg09321019;cg22591964;cg05545635 | 0.01129 |
| 2059 | 0.088209 | 0.023168 | 0.00014 | 2 | ;cg06012872;cg20638675 | 0.012044 |
| 426 | 0.068636 | 0.018103 | 0.00015 | 3 | ;cg07726288;cg08121453;cg06362358 | 0.012576 |
| 3195 | 0.067909 | 0.01794 | 0.000153 | 4 | ;cg07102001;cg04324917;cg07721872;cg00227665 | 0.012633 |
| 1630 | 0.046122 | 0.012242 | 0.000165 | 4 | ;cg15134787;cg07496173;cg03313088;cg01989621 | 0.013101 |
| 3584 | 0.076242 | 0.020257 | 0.000167 | 2 | ;cg25584814;cg08617160 | 0.013101 |
| 2507 | -0.24546 | 0.06525 | 0.000169 | 2 | ;cg26654770;cg01201512 | 0.013101 |
| 2459 | 0.069781 | 0.018704 | 0.000191 | 2 | ;cg06978117;cg03554573 | 0.01455 |
| 2097 | 0.051348 | 0.013808 | 0.0002 | 3 | ;cg27102629;cg04359418;cg14906690 | 0.014953 |
| 1628 | 0.060377 | 0.016254 | 0.000203 | 3 | ;cg16491960;cg01104724;cg20897830 | 0.014953 |
| 2229 | 0.060546 | 0.016377 | 0.000218 | 4 | ;cg12047941;cg25050332;cg08000731;cg15013527 | 0.01576 |
| 1284 | -0.05299 | 0.014355 | 0.000223 | 2 | ;cg20112500;cg11934771 | 0.015811 |
| 1051 | -0.04477 | 0.012154 | 0.00023 | 4 | ;cg06998965;cg17019292;cg05901765;cg06418871 | 0.016053 |
| 1520 | -0.0549 | 0.014971 | 0.000245 | 3 | ;cg02827175;cg16993108;cg01952989 | 0.016829 |
| 1166 | 0.092011 | 0.025198 | 0.000261 | 2 | ;cg18256205;cg10430077 | 0.017587 |
| 2177 | 0.068544 | 0.018842 | 0.000275 | 2 | ;cg09953287;cg11320749 | 0.018197 |
| 1946 | 0.035074 | 0.00965 | 0.000279 | 2 | ;cg08578641;cg23579062 | 0.018197 |
| 2135 | 0.031411 | 0.008674 | 0.000293 | 3 | ;cg21378403;cg14585415;cg05593775 | 0.018497 |
| 733 | 0.073311 | 0.020261 | 0.000297 | 6 | ;cg19875656;cg02834909;cg19668234;cg17460445;cg01967580;cg05727070 | 0.018497 |
| 2444 | 0.093399 | 0.025839 | 0.000301 | 3 | ;cg09610772;cg18725375;cg15146462 | 0.018497 |
| 857 | -0.08438 | 0.023383 | 0.000308 | 2 | ;cg06305891;cg10959668 | 0.018497 |

| | | | | | | |
|---|---|---|---|---|---|---|
| 4091 | 0.105677 | 0.029286 | 0.000308 | 2 | ;cg12301347;cg15236483 | 0.018497 |
| 3534 | 0.053224 | 0.014757 | 0.00031 | 2 | ;cg12327405;cg06595595 | 0.018497 |
| 1516 | 0.053157 | 0.014763 | 0.000317 | 2 | ;cg26580761;cg09367467 | 0.018657 |
| 1737 | -0.04829 | 0.013452 | 0.000331 | 3 | ;cg02711872;cg20142972;cg14711997 | 0.018837 |
| 2611 | 0.046313 | 0.012907 | 0.000333 | 3 | ;cg14573817;cg04686545;cg10082647 | 0.018837 |
| 77 | 0.050468 | 0.014068 | 0.000334 | 2 | ;cg12258811;cg14204433 | 0.018837 |
| 2676 | 0.056208 | 0.01572 | 0.000349 | 4 | ;cg07775468;cg15962965;cg06128161;cg01577029 | 0.019233 |
| 3735 | 0.052361 | 0.014662 | 0.000355 | 6 | ;cg06710596;cg15729137;cg25681177;cg25498045;cg01443452;cg15426035 | 0.019233 |
| 645 | 0.050609 | 0.01418 | 0.000358 | 2 | ;cg17009069;cg02231590 | 0.019233 |
| 3721 | 0.126307 | 0.035401 | 0.00036 | 3 | ;cg04546413;cg12756686;cg03161606 | 0.019233 |
| 3647 | -0.06261 | 0.017613 | 0.000378 | 2 | ;cg02346997;cg22322184 | 0.019957 |
| 823 | 0.068237 | 0.019246 | 0.000392 | 2 | ;cg19027710;cg21597025 | 0.020036 |
| 1808 | 0.034339 | 0.00969 | 0.000394 | 4 | ;cg02601352;cg04302570;cg12131827;cg26846409 | 0.020036 |
| 590 | -0.03049 | 0.008605 | 0.000394 | 2 | ;cg21303039;cg00133811 | 0.020036 |
| 3138 | 0.015008 | 0.00424 | 0.000401 | 6 | ;cg06877200;cg04436255;cg09137696;cg18438944;cg02263813;cg00425710 | 0.020138 |
| 861 | 0.061115 | 0.017308 | 0.000414 | 2 | ;cg05542785;cg11867616 | 0.020534 |
| 471 | 0.051111 | 0.014507 | 0.000426 | 2 | ;cg15423357;cg01884057 | 0.020886 |
| 2469 | 0.056517 | 0.01612 | 0.000455 | 2 | ;cg26706187;cg04475687 | 0.022022 |
| 1398 | -0.05394 | 0.015475 | 0.000492 | 2 | ;cg09861824;cg04911669 | 0.023527 |
| 1450 | 0.029408 | 0.008449 | 0.0005 | 2 | ;cg00788177;cg21583723 | 0.023587 |
| 1480 | 0.087587 | 0.025185 | 0.000506 | 2 | ;cg21205282;cg22883453 | 0.023587 |
| 2675 | 0.051402 | 0.01479 | 0.00051 | 2 | ;cg22977246;cg09698465 | 0.023587 |
| 1527 | 0.087048 | 0.025117 | 0.000529 | 2 | ;cg19346786;cg15247329 | 0.024186 |
| 3588 | 0.096623 | 0.028073 | 0.000578 | 2 | ;cg06595479;cg21450784 | 0.02613 |
| 2285 | -0.07644 | 0.022276 | 0.0006 | 3 | ;cg06005892;cg25627263;cg10803034 | 0.026848 |
| 3658 | 0.024378 | 0.007123 | 0.00062 | 2 | ;cg18072388;cg00827581 | 0.027445 |
| 1229 | 0.072233 | 0.021157 | 0.00064 | 8 | ;cg16731266;cg07017437;cg27535677;cg22497095;cg26865747;cg20851828;cg14654363;cg00990380 | 0.027589 |
| 3405 | 0.054952 | 0.016123 | 0.000653 | 2 | ;cg24992471;cg17129388 | 0.027589 |
| 434 | 0.054306 | 0.015945 | 0.00066 | 4 | ;cg05422049;cg05854217;cg10522607;cg03550129 | 0.027589 |
| 2872 | 0.072981 | 0.021435 | 0.000662 | 4 | ;cg24127506;cg23012310;cg23429749;cg15444358 | 0.027589 |
| 2254 | 0.038579 | 0.011342 | 0.00067 | 2 | ;cg02376282;cg08373904 | 0.027589 |
| 1139 | 0.031933 | 0.009389 | 0.000671 | 3 | ;cg07292251;cg24185628;cg12477903 | 0.027589 |
| 1181 | 0.042267 | 0.012433 | 0.000675 | 2 | ;cg10091752;cg13318241 | 0.027589 |
| 605 | 0.068754 | 0.020235 | 0.000679 | 3 | ;cg15602054;cg17598339;cg01268571 | 0.027589 |
| 2256 | -0.02776 | 0.008174 | 0.000684 | 2 | ;cg26241460;cg10900641 | 0.027589 |
| 2152 | 0.048729 | 0.014394 | 0.000711 | 2 | ;cg20925954;cg06110816 | 0.028409 |
| 3508 | 0.05967 | 0.017644 | 0.00072 | 2 | ;cg01763666;cg18209359 | 0.028498 |

| | | | | | | |
|---|---|---|---|---|---|---|
| 3147 | 0.136423 | 0.040379 | 0.000729 | 5 | ;cg05333442;cg27113419;cg05725404;cg00758881;cg04484415 | 0.028562 |
| 1730 | 0.034565 | 0.010239 | 0.000736 | 2 | ;cg11060441;cg02906407 | 0.028585 |
| 2470 | 0.048935 | 0.014551 | 0.000771 | 3 | ;cg27362048;cg09044186;cg25454270 | 0.029449 |
| 470 | 0.053044 | 0.015776 | 0.000773 | 3 | ;cg01343041;cg18587476;cg17277199 | 0.029449 |
| 743 | 0.055091 | 0.016473 | 0.000825 | 4 | ;cg02222844;cg08640901;cg15142694;cg09397359 | 0.030802 |
| 197 | -0.05032 | 0.015054 | 0.000829 | 2 | ;cg07150145;cg02291556 | 0.030802 |
| 1636 | 0.056188 | 0.016811 | 0.000831 | 5 | ;cg08964643;cg18313661;cg21733531;cg17662941;cg26105076 | 0.030802 |
| 3285 | 0.05263 | 0.01584 | 0.000892 | 2 | ;cg04157161;cg12504247 | 0.032622 |
| 3601 | -0.0554 | 0.016687 | 0.0009 | 4 | ;cg11683663;cg02487452;cg08223924;cg19628469 | 0.032622 |
| 3779 | 0.049579 | 0.014938 | 0.000904 | 3 | ;cg19463256;cg19747604;cg17375267 | 0.032622 |
| 1620 | 0.08858 | 0.026728 | 0.000919 | 2 | ;cg21540359;cg03689146 | 0.032768 |
| 4104 | 0.042809 | 0.012925 | 0.000926 | 3 | ;cg12961733;cg07774777;cg18232772 | 0.032768 |
| 2752 | 0.05751 | 0.017372 | 0.000931 | 5 | ;cg05912299;cg14972141;cg16665310;cg20066226;cg13752184 | 0.032768 |
| 3558 | 0.034936 | 0.0106 | 0.000982 | 3 | ;cg16004501;cg19533231;cg16439118 | 0.034092 |
| 3533 | 0.04132 | 0.012542 | 0.000986 | 2 | ;cg15648792;cg05331731 | 0.034092 |
| 3770 | -0.02607 | 0.007952 | 0.001044 | 3 | ;cg17502434;cg02360514;cg00314427 | 0.035793 |
| 3505 | -0.0481 | 0.014715 | 0.00108 | 2 | ;cg02280226;cg06483978 | 0.036309 |
| 3303 | 0.078214 | 0.023938 | 0.001085 | 5 | ;cg04306489;cg14078639;cg03049249;cg04316233;cg02879122 | 0.036309 |
| 1850 | 0.071468 | 0.021881 | 0.00109 | 2 | ;cg04889069;cg08415582 | 0.036309 |
| 222 | 0.014901 | 0.004564 | 0.001094 | 2 | ;cg18116902;cg26347197 | 0.036309 |
| 3102 | 0.025928 | 0.007956 | 0.001119 | 3 | ;cg05433111;cg01758575;cg14102807 | 0.036833 |
| 2990 | 0.039817 | 0.012238 | 0.00114 | 4 | ;cg27045999;cg18837035;cg18028999;cg09236163 | 0.037123 |
| 1174 | 0.070945 | 0.021815 | 0.001145 | 2 | ;cg07515565;cg27095915 | 0.037123 |
| 2326 | 0.054081 | 0.016654 | 0.001165 | 2 | ;cg07298299;cg05265359 | 0.037453 |
| 2327 | 0.051387 | 0.015875 | 0.001208 | 2 | ;cg20068058;cg22902089 | 0.038006 |
| 3060 | 0.076417 | 0.023631 | 0.001222 | 8 | ;cg18345369;cg00699901;cg21452411;cg27620871;cg02256969;cg19328294;cg05895034;cg09745688 | 0.038006 |
| 2827 | 0.081532 | 0.025235 | 0.001234 | 3 | ;cg23092040;cg19641841;cg22789982 | 0.038006 |
| 1944 | 0.046752 | 0.014479 | 0.001242 | 2 | ;cg11993322;cg13811417 | 0.038006 |
| 651 | 0.063164 | 0.019572 | 0.00125 | 4 | ;cg24767131;cg25560398;cg17204652;cg09490371 | 0.038006 |
| 1629 | 0.0739 | 0.022901 | 0.001252 | 9 | ;cg03829739;cg03122674;cg24398379;cg13763098;cg26262573;cg17328716;cg20115597;cg27550060;cg01922095 | 0.038006 |
| 3088 | 0.058854 | 0.018244 | 0.001255 | 3 | ;cg03014241;cg10784813;cg04004558 | 0.038006 |
| 3688 | 0.054976 | 0.017048 | 0.001261 | 2 | ;cg18473733;cg02668248 | 0.038006 |
| 2842 | 0.037138 | 0.01152 | 0.001265 | 2 | ;cg13497903;cg08108868 | 0.038006 |
| 3132 | 0.090414 | 0.028118 | 0.001302 | 5 | ;cg02382016;cg08615070;cg08681685;cg10144400;cg04506728 | 0.038631 |
| 1499 | -0.02866 | 0.008919 | 0.001314 | 3 | ;cg13477101;cg27454412;cg07217954 | 0.038631 |

| | | | | | | |
|---|---|---|---|---|---|---|
| 126 | 0.06081 | 0.018935 | 0.00132 | 2 | ;cg01519464;cg25140783 | 0.038631 |
| 730 | 0.072601 | 0.022645 | 0.001346 | 2 | ;cg04459086;cg17683948 | 0.038631 |
| 384 | 0.061995 | 0.019338 | 0.001347 | 10 | ;cg21436055;cg22344745;cg15594585;cg20011248;cg18947553;cg27056132;cg02736395;cg05053440;cg14789818;cg20699586 | 0.038631 |
| 2765 | 0.118944 | 0.037106 | 0.001348 | 2 | ;cg00557959;cg15756319 | 0.038631 |
| 107 | 0.052246 | 0.016302 | 0.001352 | 2 | ;cg06662140;cg23281527 | 0.038631 |
| 361 | 0.056928 | 0.017774 | 0.001361 | 3 | ;cg06949439;cg01442843;cg02035751 | 0.038631 |
| 2756 | -0.04603 | 0.014405 | 0.001395 | 3 | ;cg14677983;cg08400494;cg12423658 | 0.039333 |
| 1736 | 0.033125 | 0.010385 | 0.001424 | 5 | ;cg01559770;cg21737698;cg11857646;cg17402325;cg05998486 | 0.03941 |
| 2066 | 0.034876 | 0.010934 | 0.001425 | 2 | ;cg27519330;cg02616604 | 0.03941 |
| 1029 | 0.023365 | 0.007327 | 0.001429 | 3 | ;cg15758008;cg25592564;cg05999195 | 0.03941 |
| 3105 | 0.053498 | 0.016785 | 0.001436 | 2 | ;cg27202236;cg05645661 | 0.03941 |
| 3514 | 0.043663 | 0.013725 | 0.001466 | 5 | ;cg02473123;cg19914554;cg20186396;cg11294761;cg25662857 | 0.039958 |
| 896 | 0.054547 | 0.017192 | 0.00151 | 2 | ;cg10635092;cg13688770 | 0.040886 |
| 3546 | 0.061842 | 0.019511 | 0.001526 | 2 | ;cg21235532;cg07669403 | 0.040903 |
| 1482 | 0.09322 | 0.029417 | 0.00153 | 2 | ;cg19406113;cg16590582 | 0.040903 |
| 1077 | 0.042854 | 0.01357 | 0.001589 | 2 | ;cg15574301;cg20385216 | 0.042185 |
| 451 | 0.090329 | 0.028656 | 0.001621 | 2 | ;cg09590377;cg21917740 | 0.04276 |
| 3167 | -0.0616 | 0.019553 | 0.001631 | 3 | ;cg03991512;cg07320140;cg09899215 | 0.042763 |
| 395 | 0.053605 | 0.017056 | 0.001673 | 2 | ;cg27524192;cg19631585 | 0.043593 |
| 1185 | 0.085331 | 0.02725 | 0.00174 | 2 | ;cg24920126;cg10563109 | 0.045037 |
| 1162 | 0.028331 | 0.009057 | 0.001759 | 4 | ;cg05720159;cg19801705;cg25604183;cg04645331 | 0.045245 |
| 3758 | 0.034452 | 0.011031 | 0.001789 | 2 | ;cg26200585;cg01447828 | 0.045731 |
| 1257 | 0.042296 | 0.01355 | 0.0018 | 4 | ;cg13196879;cg19382642;cg00354641;cg13436843 | 0.045731 |
| 2166 | -0.09012 | 0.028926 | 0.001836 | 2 | ;cg05971678;cg11803859 | 0.046147 |
| 3668 | -0.0334 | 0.010723 | 0.001841 | 10 | ;cg26772540;cg25394203;cg26590664;cg21771200;cg15209566;cg02274869;cg10123421;cg10608596;cg00965023;cg24159514 | 0.046147 |
| 1295 | 0.066993 | 0.021518 | 0.00185 | 3 | ;cg04825215;cg11964823;cg12001709 | 0.046147 |
| 1999 | 0.042774 | 0.013748 | 0.001862 | 3 | ;cg00562504;cg02594677;cg24461627 | 0.046169 |
| 140 | 0.042594 | 0.013703 | 0.001881 | 6 | ;cg23989110;cg11592082;cg09230763;cg11705208;cg26038649;cg02673986 | 0.046333 |
| 1481 | 0.108748 | 0.03501 | 0.001895 | 2 | ;cg04386759;cg00893488 | 0.046333 |
| 266 | 0.033849 | 0.010907 | 0.001912 | 2 | ;cg13494933;cg06872981 | 0.046333 |
| 3445 | -0.06276 | 0.020222 | 0.001914 | 2 | ;cg09253696;cg04442638 | 0.046333 |
| 2466 | 0.041595 | 0.013425 | 0.001946 | 7 | ;cg20587970;cg27661571;cg06953577;cg09517809;cg26945670;cg18517222;cg18935108 | 0.046842 |
| 566 | -0.0484 | 0.015689 | 0.002037 | 2 | ;cg13700939;cg06202737 | 0.048737 |
| 2443 | 0.047555 | 0.01544 | 0.00207 | 2 | ;cg01202519;cg03102841 | 0.048985 |
| 196 | -0.04751 | 0.015425 | 0.002071 | 3 | ;cg07347511;cg00950809;cg05376185 | 0.048985 |

| | | | | | | |
|---|---|---|---|---|---|---|
| 2063 | 0.039721 | 0.012904 | 0.002083 | 4 | ;cg01464748;cg24302529;cg09832911;cg26291600 | 0.048985 |
| 2062 | 0.055467 | 0.018033 | 0.002099 | 4 | ;cg20447730;cg01782066;cg23771956;cg04392266 | 0.049077 |
| 2546 | 0.037432 | 0.012181 | 0.002118 | 5 | ;cg07634645;cg17768691;cg11905611;cg19725903;cg25642234 | 0.049114 |
| 491 | -0.03934 | 0.012805 | 0.002124 | 2 | ;cg00360761;cg00055073 | 0.049114 |
| 4053 | 0.027 | 0.0088 | 0.002153 | 2 | ;cg00093544;cg02923264 | 0.049483 |
| 3896 | 0.044907 | 0.014643 | 0.002164 | 7 | ;cg27413508;cg12033622;cg26070540;cg17116120;cg21543589;cg08918020;cg11704513 | 0.049483 |
| 1552 | -0.06332 | 0.020665 | 0.002184 | 2 | ;cg04682905;cg11554295 | 0.049517 |
| 75 | -0.02074 | 0.00677 | 0.00219 | 2 | ;cg10055231;cg12690996 | 0.049517 |
| 2918 | 0.05459 | 0.01784 | 0.002213 | 3 | ;cg09088406;cg13159361;cg07521193 | 0.049783 |

**Supplemental table 3:** List of robust enhancers from Andersson et al, 2014, which were located 300 bps within a DMC.

| Chromosome | start | end | Cluster no. | score | Max dist. | Feature location |
|---|---|---|---|---|---|---|
| chr15 | 31372997 | 31373178 | 2929 | 0.072807 | 191 | overlapEnd |
| chr2 | 2.4E+08 | 2.4E+08 | 672 | 0.130861 | 15 | inside |
| chr2 | 8597389 | 8597923 | 451 | 0.090329 | 237 | overlapEnd |
| chr3 | 1.96E+08 | 1.96E+08 | 857 | -0.08438 | 18 | inside |
| chr3 | 1.96E+08 | 1.96E+08 | 861 | 0.061115 | 197 | downstream |
